# Lifestyle recommendations and pelvic floor muscle training with Knack maneuver for post-prostatectomy urinary incontinence: a randomized controlled trial

**DOI:** 10.1007/s00520-025-09197-z

**Published:** 2025-01-31

**Authors:** Ege Nur Atabey Gerlegiz, Türkan Akbayrak, Ceren Gürşen, Mustafa Sertaç Yazici, Naşide Mangir Bolat, Bülent Akdoğan, Gülbala Nakip, Serap Özgül

**Affiliations:** 1 Faculty of Physical Therapy and Rehabilitation, Department of Fundamental Physiotherapy and Rehabilitation, Hacettepe University, Ankara, Turkey; 2 School of Medicine, Department of Urology, Hacettepe University, Ankara, Turkey

**Keywords:** Radical prostatectomy, Urinary Incontinence, Conservative treatment, Physiotherapy, Functional pelvic floor muscle training, Knack maneuver, Lifestyle change

## Abstract

**Purpose:**

The aim of this study is to investigate the additional effects of the Knack maneuver and comprehensive lifestyle recommendations to pelvic floor muscle training (PFMT) in individuals with post-prostatectomy urinary incontinence (PP-UI).

**Methods:**

Seventy-one individuals with symptom of PP-UI were included. Individuals were randomly assigned to study groups (Group I: PFMT + Knack + Comprehensive Lifestyle Recommendations, Group II: PFMT + Knack, Group III: PFMT alone). Assessments were performed at the baseline and at the end of the 8th week. The primary outcome was the subjective severity and impact of UI. Secondary outcomes were objective severity of UI, health-related quality of life (QoL) and patient global impression of severity and improvement. Descriptive and outcome measures were compared between study groups using the Kruskal–Wallis test. The Games-Howell post hoc test was also used to indicate which groups differ.

**Results:**

A total of 66 patients were included in the final analysis. Per protocol analysis in all three groups showed significant improvements in all primary and secondary outcomes in eight weeks. The group of patients who had the PFMT + Knack + Comprehensive Lifestyle Recommendations had the greatest improvement in all outcome measures (*p* < 0.001). In addition, while PFMT + Knack showed superiority in terms of subjective UI severity and effect of UI on daily life, compared to PFMT alone (*p* < 0.001), there was no inter-group differences for objective UI severity and other subdomains of QoL (*p* > 0.05).

**Conclusion:**

Adding comprehensive lifestyle recommendations and/or Knack maneuver to traditional PFMT is more effective in the management of post-prostatectomy UI in the short term. Further long-term follow-up studies should be planned to investigate compliance and response to these combined interventions.

**Clinical Trial Registration Number:**

ClinicalTrials.gov NCT04804839. Date of registration: 03/17/2021.

**Supplementary Information:**

The online version contains supplementary material available at 10.1007/s00520-025-09197-z.

## Introduction

Prostate cancer is the second most common cancer in men after lung cancer [1]. Radical prostatectomy (RP) is the gold standard surgical technique for preventing metastasis in localized prostate cancer [2]. However, RP is associated with various complications and urinary incontinence (UI) is one of the most common complications [2]. Research shows that UI rates can reach 87% in the early post-RP period and the severity of UI during this period is mostly moderate to severe [3].

Post-prostatectomy urinary incontinence (PP-UI) significantly reduces the quality of life of individuals in terms of physical, psychological, and social aspects, and therefore, individuals seek treatment for UI [4]. The management of UI after RP includes both conservative and surgical techniques. However, conservative therapies are recommended in the first-line management of female and male UI. Conservative therapies for PP-UI are recommended to be applied within the first 6–12 months following surgery. Invasive surgical interventions are recommended for patients with moderate-severe SUI for more than a year. In the first-line management, lifestyle changes or pelvic floor muscle training (PFMT) are commonly used conservative approaches [5, 6].

Healthcare professionals commonly recommend lifestyle modifications in the management of PP-UI and other health problems, because they are cost-effective and have minimal side effects. According to the comprehensive literature search, lifestyle modifications recommended in PP-UI consist of adjusting fluid intake, having a healthy diet, avoiding excessive caffeine or alcohol consumption, increasing physical activity level, gaining regular exercise habits, maintaining weight control, preventing constipation and straining, avoiding heavy lifting, taking measures to prevent urinary tract infections, and reducing/quitting smoking [7–9]. However, studies have not comprehensively address these lifestyle recommendations in the management of PP-UI, and the available evidence on the effects of restricted recommendations (e.g., fluid intake only or weight control) is extremely limited [10].

According to the guidelines, PFMT should be considered the first-line treatment for post-operative UI in all patients undergoing RP, and it should be started as soon as possible [11]. A systematic review and meta-analysis published in 2018 stated that PFMT in the management of PP-UI may provide continence earlier, but does not make a difference in long-term continence rates [12]. A systematic review and meta-analysis published in 2022 reported that supervised and high intensity PFMT is more effective than unsupervised and low-intensity training in the management of PP-UI, especially in the first 3 months after surgery [13]. On the other hand, in studies investigating the effect of PFMT on PP-UI, the focus of training is mostly on improving muscle strength, and the number of studies focusing on muscular endurance is limited. It has also been stated that the failure of PFMT on male UI can be attributed to the early or sole focus on strength training [14]. Since the pelvic floor muscle strength is generally not the main issue in post-prostatectomy UI, maximal strength or rapid contractions should not be the sole training focus. Slow and tonic holds are also required from the striated muscles to cope with the loss of the smooth muscles of the urethral sphincter [15]. In addition, strength training alone is not sufficient to prevent unexpected urine leakage during activities that cause high intra-abdominal pressure increases. Therefore, the ability of pelvic floor muscles to activate quickly is also necessary [14]. Therefore, men need to be encouraged to contract the pelvic floor muscles in anticipation of a leak occurring until the automatic phase of motor learning is reached [16]. At this point, Knack maneuver training, which is the conscious pre-contraction training of the pelvic floor muscles, seem to be a good solution. This maneuver teaches the ability to voluntarily activate the pelvic floor muscles to prevent UI during activities that increase intra-abdominal and therefore intravesical pressure. In the management of female stress urinary incontinence, it is recommended that the Knack maneuver be taught to women in addition to strength and endurance training of the pelvic floor muscles [17]. However, to our knowledge, there are no studies on whether this maneuver provides an effect on male incontinence, alone or as an additive to other therapies.

Based on the literature given above, the aim of this study was to reveal the effects of the Knack maneuver and comprehensive lifestyle recommendations program to be given in addition to the supervised PTMT program in a randomized controlled design, with the hypothesis of achieving better results in individuals suffering from PP-UI.

Our primary hypothesis was that comprehensive lifestyle recommendations combined with PFMT including the Knack maneuver would be more effective than PFMT alone (with or without the Knack maneuver) in improving incontinence severity and quality of life in men with postprostatectomy UI. Our secondary hypothesis was that PFMT with Knack maneuver would be more effective than PFMT without Knack maneuver in improving incontinence severity and quality of life.

## Methods

### Study design

This is a randomised controlled study with 3 parallel arms. In the first study arm, a comprehensive lifestyle recommendations programme was given in addition to the PFMT programme including the Knack maneuver. In the second study arm, only PFMT was given with the Knack maneuver. The third study arm included PFMT without the Knack maneuver (Group I: PFMT with Knack maneuver + lifestyle recommendations, Group II: PFMT with Knack maneuver, Group III-Control Group: Traditional PFMT without Knack maneuver).

The study protocol was approved by Hacettepe University, Clinical Research Ethics Board (Protocol code: KA-20081) and registered at clinicaltrials.gov (NCT04804839). All individuals were informed on the basis of the Declaration of Helsinki and all of them provided informed signed consent. The study was reported in accordance with the CONSORT guidelines.

### Participants

The study included men who underwent radical prostatectomy for localized prostate cancer and had symptoms of stress UI or stress-dominant mixed UI following prostatectomy. Patients underwent either robotic or open surgeries at the Hacettepe University School of Medicine Department of Urology between April 2021 and March 2022. Patients who were at least 3 weeks after catheter removal and able to voluntarily contract their pelvic floor muscles were included in the study. The ability of individuals to perform these contractions was assessed by a researcher with 5 years of experience in pelvic floor health (ENA). In addition, in terms of cooperation in evaluation and interventions, Mini Mental Test was applied to individuals and those with a test score ≥ 24 were included in the study [18].

Exclusion criteria were determined as the presence of urinary tract infection, respiratory tract infection, interstitial cystitis, acute surgical condition (within the first 3 weeks after prostatectomy), neurological disease or neurogenic bladder, pure urgency UI or urgency-dominant MUI, preoperative incontinence, bladder or other prostate surgeries before prostatectomy, or receiving PFMT within the last 6 months.

### Randomization

Participants were randomized to the groups using an online block randomization list, consisting of blocks of 6. The randomization list was created by an independent researcher (S.Ö.) who was not involved in the evaluation and intervention processes of the study.

### Interventions

#### Pelvic floor muscle training

In the first session of the PFMT, a correct pelvic floor muscle contraction was taught or confirmed by perineal/anal inspection and subsequent digital rectal palpation. The participants were assessed in a left lateral position with their legs pulled up, and the anal resting tone, voluntary contraction-relaxation capability of the pelvic floor, and reflex muscle contraction and relaxation during coughing and straining were evaluated [19]. Verbal instructions were used to elicit fast and strong contractions of the pelvic floor muscles. The most common instruction used was “squeeze and pull in.” To help participants understand the contraction, additional instructions were given, such as “squeeze as if stopping urination,” “pull your penis inward,” “pull your penis toward yourself,” “lift your scrotum and pull your penis toward your abdomen,” or “shorten your penis” [20]. Once the correct contraction technique was taught, the participants were instructed on slow-fast maximal voluntary contractions and repeated submaximal contractions to improve the strength and endurance of the pelvic floor muscles. Participants continued PFMT as a home exercise program. One set of exercises included 10 repetitions of fast (1 s contraction-1 s relaxation) and slow (slow contraction- maintaining maximum contraction for 3–6 s-slow relaxation) maximal voluntary contractions and 20 repetitions of submaximal voluntary contractions (50% of maximal contraction) to improve strength and static/dynamic endurance of pelvic floor. For the first 2 weeks of PFMT, three exercise sessions per day were prescribed. The number of sets was increased by one set during every 2-week clinical control session (week 3–4: 4 sessions, week 5–6: 5 sessions, and week 7–8: 6 sessions of exercise per day). An exercise diary was provided to all participants to track their progress, and they were instructed to mark their exercises on this diary.

#### Knack maneuver in pelvic floor muscle training

Participants in the first and second study groups received “Knack maneuver” training during for activities of daily living (coughing, sneezing, laughing, walking up and down stairs, jumping, running, bending, standing, etc.) in the clinic in addition to PFMT whose protocol is described above [17]. Patients were trained to perform the Knack maneuver through a of supervised sessions, where they received detailed instructions and feedback to ensure proper technique and efficacy. To ensure effective learning and integration into daily life, participants were advised to perform the Knack maneuver every time throughout the day, particularly before or during activities of daily living mentioned above that increase intra-abdominal pressure.

#### Recommendations for lifestyle modifications

Individuals in Group I (PFMT + Knack + Lifestyle recommendations) received comprehensive training on lifestyle modification recommendations and a written document containing these recommendations was provided. Lifestyle modifications in our study were based on individualized assessments and discussions with the participants. Each participant underwent a personalized evaluation where their specific lifestyle factors, health conditions, and preferences were taken into account. Based on these assessments, tailored recommendations were provided and discussed in detail with each participant to ensure understanding and adherence. Additionally, participants were given a booklet that outlined the general principles and guidelines for lifestyle modifications discussed during their individual assessments. Recommendations in this training included: I. management of medical conditions affecting the urinary system, II. fluid intake/diet modification, III. recommendations on smoking, IV. recommendations on physical activity/exercise, and V. recommendations on physical effort and strain [8–10].

### Descriptive and outcome measures

Demographic, physical, medical/surgical and lifestyle characteristics of the participants were recorded. Outcome measurements were conducted at the beginning of the study and at the end of the 8th week under the supervision of a blind investigator (G.N.). In addition, subjective perception of improvement in UI and adherence with PFMT were evaluated at the end of the 8th week.

I. Primary outcome measure: subjective incontinence severity and the impact of incontinence on daily life

Subjective UI severity and the impact of UI on daily life were assessed with the International Consultation on Incontinence Questionnaire-Short Form (ICIQ-UI SF) [21, 22]. The Turkish ICIQ-UI SF was shown to have good internal consistency (Cronbach α = 0.71) and high test–retest reliability (0.95–0.98) [21]. A higher score of the questionnaire indicates an increase in the severity of incontinence and the effect of incontinence on daily life.

II. Secondary outcome measures

- Objective incontinence severity
  The severity of incontinence was objectively evaluated by the 1-h pad test, which is a widely used, cost-effective, and non-invasive test for the objective assessment of the severity of UI [23, 24]. The pad test aims to quantify urinary leakage by measuring the weight difference of incontinence products before and after a series of provocation maneuvers. All participants were provided with pre-weighed pads to replace any pads they were already wearing. Each participant was instructed to drink 500 ml of plain water over 15 min. The next 30 min involved walking around. During the final 15 min, participants were instructed to perform the standard ICS provocation exercises in a private area [25]. Afterward, the pads were re-weighed using a gram-sensitive scale to measure the amount of urinary leakage.
- The impact of incontinence on quality of life sub-domains
  King’s Health Questionnaire (KHQ) was used to investigate the impact of interventions on the sub-domains of quality of life [26, 27]. The Turkish-KHQ has good internal consistency (Cronbach α = 0.68) and test–retest reliability (0.72–0.89) [26].
- Patient global impression of severity
  The severity impression of UI was recorded as 1: Normal, 2: Mild, 3: Moderate, and 4: Severe using the “Patient Global Impression of Severity Scale” [28, 29].
- Patient global impression of improvement
  Participants’ impression of improvement in UI complaints was recorded in 7 grades ranging from “much better” to “much worse” using the “Patient Global Impression of Improvement Scale” [28, 29].
- Adherence to lifestyle recommendations and pelvic floor exercises
  Compliance with the lifestyle training, which included recommendations for consumption of water, tea, coffee, sugary/carbonated drinks and taking alcohol, smoking and bowel pattern, was evaluated with Likert-type scales. In addition, physical activity levels of the participants were assessed with the Turkish-International Physical Activity Questionnaire-Short Form (IPAQ-SF) [30].
  The participants’ adherence to lifestyle recommendations and pelvic floor exercises was calculated as a percentage (%) based on the lifestyle recommendations diary and exercise diary data sheet given to the participants.
- Adverse events
  Adverse events were systematically assessed to identify and document potential negative effects resulting from the treatment. All participants were regularly monitored for adverse events throughout the treatment period and follow-up visits.

### Data analyses

Data analysis was conducted using SPSS 25.0 (IBM SPSS, Armonk, NY, USA) software. The parametric/nonparametric distribution of the data was analyzed with the Kolmogorov–Smirnov test. Numerical data were presented as mean and SD, and categorical data were presented as frequency (*n*) and percentage (%). The Kruskal–Wallis test was used to compare descriptive and outcome measures between the study groups. In case of a significant difference between the groups, the Games Howell Post-Hoc test was used to determine the groups where the difference originated. The Wilcoxon test was used for within-group comparisons of outcomes. The Chi-square test or Fisher’s exact test was used for comparison of categorical variables between groups. We opted not to use an intention-to-treat (ITT) approach in our analyses due to practical considerations related to participant compliance and protocol deviations. Instead, we conducted per-protocol analyses to focus on participants who adhered strictly to the study protocol. Statistical significance was determined as *p* < 0.05.

### Sample size

Based on randomized controlled trials in which the ICIQ-UI SF score was the primary outcome measure, the between-group effect size for the ICIQ-UI SF score was determined as *d* = 0.4 [31–33]. In the two-way hypothesis test design, the sample size was calculated as 66 participants in total (22 participants), with 80% power and 5% type 1 margin of error. Considering a total 10% dropout rate, the final total sample size required was calculated as 72 individuals, with 24 individuals per study group.

## Results

**Fig. 1:**
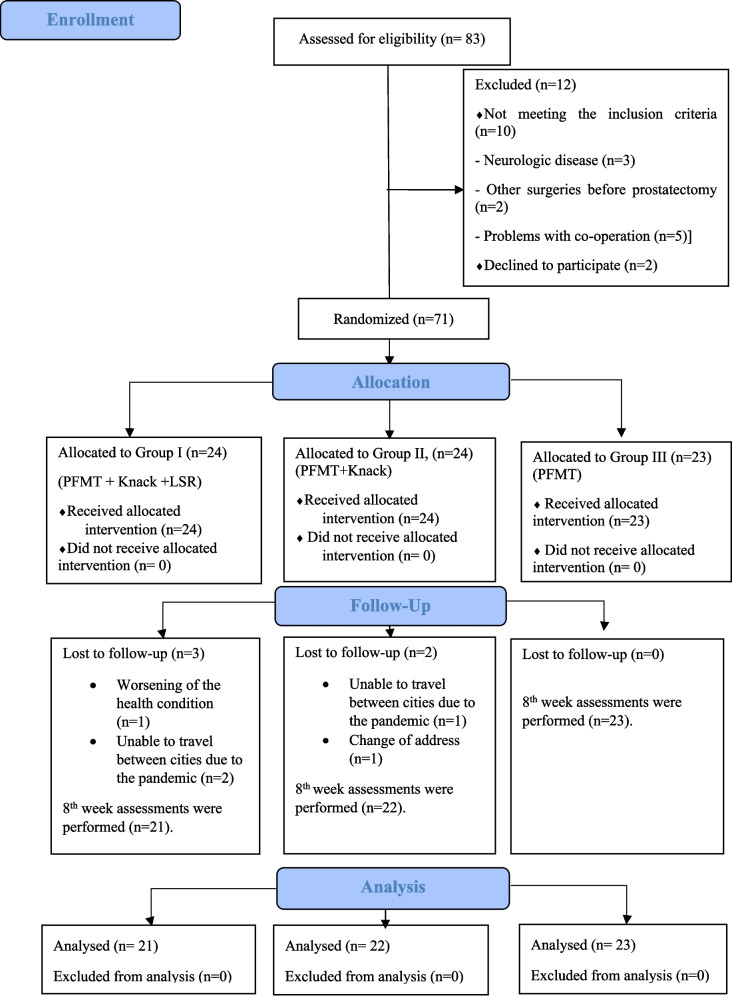
CONSORT flow diagram of the three study groups. *PFMT* pelvic floor muscle training, *LSR* lifestyle recommendations, *n* number

In this study, 83 individuals with symptoms of UI after prostatectomy were screened for inclusion criteria between April 2021 and March 2022. Ten participants did not meet the inclusion criteria and 2 individuals declined to participate in the study. Thus, 71 participants were included in the study. During the study period, 5 participants dropped out of the study and a total of 66 participants completed the study (Group I, *n* = 21; Group II, *n* = 22, and Group III, *n* = 23). Figure 1 shows the flow diagram of the participants.

At the baseline of the study, there was no difference between the study groups in terms of descriptive, primary, and secondary outcome measures (*p* > 0.05) (Tables 1 and 2).

**Table 1:** Comparison of demographic, medical, and lifestyle characteristics of groups

Parameters	Group I (*n* = 21)	Group II (*n* = 22)	Group III (*n* = 23)	*p*
**Physical and demographic characteristics**				
Age, y	62.33 ± 6.9	66 ± 8.42	64.61 ± 5.09	0.223^a^
BMI, kg/m^2^	27.78 ± 3.30	26.23 ± 3.36	27.66 ± 3.70	0.267^a^
Education, y	14.28 ± 3.86	14.5 ± 3.08	13.65 ± 3.93	0.721^a^
Marital status				
Married	19 (%28.8)	20 (%30.3)	22 (%33.3)	0.732^b^
Single	2 (%3.0)	2 (%3.0)	1 (%1.5)	
Working status				
Employed	9 (%13.6)	6 (%9.1)	10 (%15.2)	0.454^b^
Unemployed	12 (%18.2)	16 (%24.2)	13 (%19.7)	
**Medical characteristics**				
Incontinence Symptom Duration, month	2.71 ± 2.10	1.77 ± 2.34	1.32 ± 2.59	0.845^a^
Type of surgery				
Laparoscopic radical prostatectomy	13 (%19.7)	14 (%21.2)	14 (21.2)	0.982^b^
Retropubic radical prostatectomy	8 (%12.1)	8 (%12.1)	9 (%13.6)	
Gleason Score				
< 7	14 (%66.7)	16 (%72.7)	15 (%65.2)	0.850^b^
≥ 7	7 (%33.3)	6 (27.3)	8 (34.8)	
Preoperative PSA level, ng/mL	12.07 ± 9.52	11.81 ± 8.01	15.18 ± 8.90	0.419^a^
Nerve-sparing procedure				
Yes	13 (%61.9)	14 (%63.6)	14 (%60.87)	0.878^b^
No	8 (%38.1)	8 (%36.4)	9 (%39.13)	
Chronic Disease (yes)	16 (%24.2)	20 (%30.3)	18 (%27.3)	0.847^b^
Constipation (yes)	3 (%4.5)	3 (%4.5)	5 (%7.6)	0.772^b^
**Lifestyle characteristics**				
Daily water consumption, lt	1.78 ± 0.56	1.65 ± 0.58	1.46 ± 0.79	0.287^a^
Tea Consumption (yes)	21 (%31.8)	21 (%31.8)	21 (%31.8)	0.768^b^
Coffee Consumption (yes)	8 (%12.1)	13 (%19.7)	14 (%21.2)	0.250^b^
Consumption of sugary/gas drinks (yes)	2 (%3.0)	1 (%1.5)	0 (%0)	0.202^b^
Alcohol Intake (yes)	3 (%4.5)	6 (%9.1)	3 (%4.5)	0.482^b^
Smoking (yes)	4 (%6.1)	5 (%7.6)	3 (%4.5)	0.954^b^
Smoking, pack-years	23.20 ± 19.24	23.59 ± 16.33	23.20 ± 17.40	0.997^a^
Physical Activity Score, (MET X min/week)	1669.76 ± 727.95	1616.45 ± 675.95	1691 ± 710.77	0.936^a^
**IPAQ-SF level**				
Low Physical Activity	5 (%23.8)	6 (%27.3)	6 (%26.1)	0.714^b^
Moderate Physical Activity	14 (%66.7)	15 (%68.2)	13 (%56.5)	
High Physical Activity	2 (%9.5)	1 (%4.5)	4 (%17.4)	

Values are presented as mean ± standard deviation or frequency (percentage). *BMI* body mass index, *PSA* prostate specific antigen, *IPAQ*-*SF* International Physical Activity Questionnaire-Short Form, *MET* metabolic equivalent

^a^One-way analysis of variance

^b^Chi-square test

**Table 2:** Comparison of baseline values of primary and secondary outcome measurements for groups

Outcome measurement	Group I (*n* = 21)	Group II (*n* = 22)	Group III (*n* = 23)	*p*
**Primary outcome**				
Subjective Incontinence Severity and General Quality of Life Score (ICIQ-UI SF) (0–21)	14.86 ± 3.09	13.73 ± 3.45	13.91 ± 3.53	0.508^a^
**Secondary outcomes**				
Objective Incontinence Severity (1-Hour Pad Test)	37.37 ± 31.41	36.97 ± 35.46	36.91 ± 33.78	0.985^a^
Quality of Life Sub-Domains (KHQ)				
GHP (0–100)	26.19 ± 9.61	25 ± 10.91	30.43 ± 18.4	0.430^a^
II (0–100)	80.95 ± 19.92	69.7 ± 22.79	68.12 ± 23.52	0.170^a^
RL (0–100)	49.21 ± 33.94	43.18 ± 31.14	36.23 ± 35.41	0.541^a^
PL (0–100)	35.71 ± 27.02	37.12 ± 25.16	42.75 ± 24.53	0.438^a^
SL (0–100)	38.1 ± 33.99	31.31 ± 23.16	32.13 ± 28.82	0.731^a^
LPR (0–100)	36.66 ± 39.91	18.52 ± 13.03	38.46 ± 42.15	0.921^a^
EP (0–100)	30.16 ± 32.61	43.43 ± 39.83	36.71 ± 32.21	0.483^a^
SED (0–100)	46.03 ± 26.83	34.85 ± 26.18	48.55 ± 30.12	0.411^a^
SM (0–100)	35.56 ± 14.96	36.67 ± 15.22	43.77 ± 18.95	0.389^a^
Patient Impression of Severity (PGI-S)				
Normal, *n* (%)	2 (%66.7)	1 (%33.3)	0 (0.0)	0.493^b^
Mild, *n* (%)	3 (%27.3)	6 (%54.5)	2 (%18.2)	
Moderate, *n* (%)	10 (%28.6)	10 (%28.6)	15 (%42.9)	
Severe, *n* (%)	6 (%35.3)	5 (%29.4)	6 (%35.3)	

Values are presented as mean ± standard deviation or frequency (percentage)

*ICIQ*-*UI SF* International Incontinence Consultation Questionnaire-Short Form, *KHQ* King Health Questionnaire, *GHP* General Health Perception, *II* incontinence impact, *RL* role limitations, *PL* physical limitations, *SL* social limitations, *LPR* limitations in personal relationship, *EP* emotional problems, *SED* sleep and energy disturbances, *SM* severity measures, *PGI*-*S* Patient Global Impression of Severity

^a^Kruskal-Wallis test, Post-Hoc: Games Howell test

^b^Pearson Chi-Square Test: Fisher Exact Test

### Primary outcome measure: subjective incontinence severity and the impact of incontinence on daily life

ICIQ-UI SF scores showed statistically significant improvement in Group I compared to the other groups at the end of the 8th week (p_GroupI-GroupII_ = 0.024 and p_GroupI-GroupIII_ < 0.001). In addition, the amount of improvement in Group II was higher than in Group III at the end of the 8th week (p_GroupII-GroupIII_ = 0.022) (Table 3).

**Table 3:** Comparison of primary and secondary outcome measurements within and between group

Outcome measurements	Time point	Group I (*n* = 21)	Group II (*n* = 22)	Group III (*n* = 23)	*p*^a^
**Primary outcome measurement**					
**ICIQ-UI SF** (0–21)	Baseline	14.86 ± 3.09	13.73 ± 3.45	13.91 ± 3.53	0.508
	After Intervention	4.38 ± 2.80^x^	6.41 ± 1.92^y^	9.04 ± 4.03^z^	** < 0.001**
	p^b^	** < 0.001**	** < 0.001**	** < 0.001**	
**Secondary Outcome Measurements**					
**1 Hour Pad Test**	Baseline	37.37 ± 31.41	36.97 ± 35.46	36.91 ± 33.78	0.985
	After Intervention	2.47 ± 2.6^x^	9.94 ± 12.77^y^	22.24 ± 28.41^y^	**0.040**
	p^b^	** < 0.001**	** < 0.001**	** < 0.001**	
**KHQ Sub-domains**					
GHP (0–100)	Baseline	26.19 ± 9.61	25 ± 10.91	30.43 ± 18.4	0.430
	After Intervention	25 ± 17.68	28.41 ± 11.69	29.35 ± 16.26	0.452
	p^b^	0.882	0.727	0.165	
II (0–100)	Baseline	80.95 ± 19.92	69.7 ± 22.79	68.12 ± 23.52	0.170
	After Intervention	14.29 ± 19.92^x^	33.33 ± 20.57^y^	52.17 ± 28.12^z^	** < 0.001**
	p^b^	** < 0.001**	** < 0.001**	**0.017**	
RL (0–100)	Baseline	49.21 ± 33.94	43.18 ± 31.14	36.23 ± 35.41	0.541
	After Intervention	4.76 ± 7.72^x^	16.67 ± 17.06^y^	31.16 ± 31.1^y^	**0.002**
	p^b^	**0.001**	**0.002**	0.763	
PL (0–100)	Baseline	35.71 ± 27.02	37.12 ± 25.16	42.75 ± 24.53	0.438
	After Intervention	7.14 ± 12.44^x^	23.49 ± 23.94^y^	23.91 ± 29.23^y^	**0.006**
	p^b^	**0.003**	**0.034**	**0.007**	
SL (0–100)	Baseline	38.1 ± 33.99	31.31 ± 23.16	32.13 ± 28.82	0.731
	After Intervention	11.63 ± 20.92	15.66 ± 21.04	17.87 ± 31.38	0.423
	p^b^	**0.004**	**0.019**	0.174	
LPR (0–100)	Baseline	36.66 ± 39.91	18.52 ± 13.03	38.46 ± 42.15	0.921
	After Intervention	1.39 ± 4.81^x^	21.43 ± 28.06^y^	31.25 ± 40.77^y^	**0.018**
	p^b^	0.105	0.325	0.260	
EP (0–100)	Baseline	30.16 ± 32.61	43.43 ± 39.83	36.71 ± 32.21	0.483
	After Intervention	5.29 ± 10.9^x^	16.67 ± 19.92^y^	34.3 ± 31.94^y^	**0.001**
	p^b^	**0.007**	**0.003**	0.070	
SED (0–100)	Baseline	46.03 ± 26.83	34.85 ± 26.18	48.55 ± 30.12	0.411
	After Intervention	21.43 ± 20.51	28.03 ± 15.76	26.81 ± 26.47	0.547
	p^b^	**0.002**	0.257	**0.012**	
**Treatment adherence**	After Intervention	97.37 ± 4.88	97.20 ± 5.06	98.33 ± 3.04	0.984

Data are presented as mean ± standard deviation or frequency (percentage)

*ICIQ*-*UI SF* International Incontinence Consultation Questionnaire-Short Form, *KHQ* King Health Questionnaire, *GHP* General Health Perception, *II* incontinence impact, *RL* role limitations, *PL* physical limitations, *SL* social limitations, *LPR* limitations in personal relationship, *EP* emotional problems, *SED* sleep and energy disturbances

^a^Kruskal-Wallis test, Post-Hoc: Games Howell test

^b^Wilcoxon test, ^x,y,z^ = Mann–Whitney *U* test, A difference between pairs is indicated by different upper indices in the same row

### Secondary outcome measures

At the end of the 8th week, the amount of urinary leakage by 1-h pad test was significantly lower in Group I compared to those of the other study groups (*p* < 0.05) (Table 3).

At the end of the 8th week, statistically significant intergroup differences were observed in favour of the combined intervention group in the sub-domains of “incontinence impact,” “role limitations,” “physical limitations,” “limitations in personal relationships,” and “emotional problems” (*p* < 0.05) (Table 3).

According to the results of patient global impression of severity and improvement, there were statistically significant differences between groups in favour of Group I at the end of the 8th week (*p* = 0.004 and *p* = 0.001, respectively) (Table 4).

**Table 4:** Comparison of categorical secondary outcome measurements within and between group

Outcome measurements	Time point	Condition	Group I (*n* = 21)	Group II (*n* = 22)	Group III (*n* = 23)	*P*^a^
**Impression of severity**	Baseline	Normal	2 (%66.7)	1 (%33.3)	0 (%0.0)	0.493
		Mild	3 (%27.3)	6 (%54.5)	2 (%18.2)	
		Moderate	10 (%28.6)	10 (%28.6)	15 (%42.9)	
		Severe	6 (%35.3)	5 (%29.4)	6 (%35.3)	
	After Intervention	Normal	14 (%60.9)	6 (%26.1)	3 (%13.0)	**0.004**
		Mild	5 (%20.0)	9 (%36.0)	11 (%44.0)	
		Moderate	2 (%13.3)	7 (%46.7)	6 (%40.0)	
		Severe	0 (0.0)	0 (0.0)	3 (%100.0)	
**Impression of improvement**	After Intervention	Much Better	9 (%60.0)	4 (%26.7)	2 (%13.3)	**0.001**
		Better	11 (%36.7)	12 (%40.0)	7 (%23.3)	
		A Little Better	1 (%5.0)	6 (%30.0)	13 (%65.0)	
		No change	0 (0.0)	0 (0.0)	1 (%100.0)	

Data are presented as frequency (percentage). *PGI*-*S* Patient Global Impression of Severity, *PGI*-*I* Patient Global Impression of Improvement

^a^Pearson Chi-Square Test: Fisher Exact Test

The rates of change with lifestyle recommendations in Group I ranged between 14.4 and 100%. Moreover, the average physical activity level scores of the individuals increased significantly in this group (*p* < 0.001) (Table 5). At the end of the 8th week, there was no difference between the groups in terms of adherence rate to pelvic floor exercises (*p* > 0.05) (Table 3).

**Table 5:** Distribution of level of adherence to lifestyle recommendations and physical activity score

*Adherence to Lifestyle Recommendations*		Week 8
Parameters	Adherence Level	*n*(%)
Tea consumption	I did not change	2 (9.5) 19 (90.5) 8 (38.1) 9 (42.9) 2 (9.5)
	I changed it • I reduced it a little	
	• I reduced a lot	
	• I quit	
Coffee consumption	I did not change	8 (38.1) 13 (61.9) 7 (33.3) 3 (14.3) 3 (14.3)
	I changed it • I reduced it a little	
	• I reduced a lot	
	• I quit	
Smoking	I did not change	19 (90.5) 2 (9.5)
	I reduced it a little	
Alcohol intake	I did not change	18 (85.6) 3 (14.4) 1 (4.8) 1 (4.8) 1 (4.8)
	I changed it • I reduced it a bit	
	• I reduced it a lot	
	• I quit	
Water consumption	I did not change	0 (0) 21 (100.0)
	I paid attention to the recommendation	
Sugary/carbonated drinks	I did not change	18 (85.7) 3 (14.3) 1 (4.8) 0 (0) 2 (9.5)
	I changed it • I reduced it a bit	
	• I reduced it a lot	
	• I quit	
Recommendations for constipation	I never followed I followed	17 (81.0) 4 (19) 0 (0) 2 (9.5) 2 (9.5)
	• I followed the recommendations about constipation a little	
	• I followed the recommendations about constipation very much	
	• I fully complied with the recommendations on constipation	
***Physical activity score***	Baseline	1669.76 ± 727.95
	After Intervention Period	2524.38 ± 1026.41
	*p*	** < 0.001**

*n* number, % frequency, *p* Wilcoxon test

No adverse events were reported or observed during the study.

## Discussion

The present study aimed to evaluate the effects of adding the Knack maneuver and comprehensive lifestyle training to traditional PFMT for the management of PP-UI. To our knowledge, this is the first randomized controlled trial to assess the potential benefits of this combined approach. The results indicate that adding the Knack maneuver and lifestyle recommendations to PFMT led to improvements in all outcome measures. While the addition of the only Knack maneuver training to PFMT resulted in greater improvement in terms of subjective UI severity and impact on daily life compared to PFMT alone, there was no difference for other outcome measures. Lifestyle recommendations are commonly given in the management of UI. However, these recommendations are mostly given superficially (verbally or in a printed document format) and compliance with the recommendations is not questioned. In addition, the recommendations focus on specific aspects of lifestyle (e.g., regulation of fluid intake or weight control) and the effects of the recommendations have been studied predominantly in the female population. In a Cochrane review examining the effects of lifestyle changes on UI, almost all of the individuals in the included studies were women (5974 women, 20 men) [10]. It is also stated that the effects of lifestyle modification on PP-UI are uncertain and further research is needed [34]. Furthermore, studies have demonstrated that advanced age, comorbidities, partial denervation of the bladder, and “de novo overactive bladder” may cause overactive bladder symptoms after prostatectomy [35]. Based on all this knowledge, an extensive literature search was conducted in our study and lifestyle recommendations for common potential bladder symptoms were compiled. The patients received a standardised training session which explained the recommendations and their mechanisms of action.

PFMT is recommended in the first-line management of PP-UI. PFMT improves UI and quality of life by improving pelvic floor muscle strength, endurance and coordination [36–39]. A review published in 2018 reported that PFMT accelerates the recovery of continence in individuals with UI following prostatectomy [12]. In addition, there are randomized controlled trials showing that the effect of training is maintained in the long term [36]. Since the study design, starting time, type, intensity, and duration of PFMT, and outcome measures are heterogeneous in existing studies addressing PP-UI, it is difficult to compare study results. While the duration of PFMT varies from 4 weeks to 12 months, the number pelvic floor muscle contractions per day varies between 30 and 150 [12]. In the present study, the duration of PFMT was 2 months and the training intensity was high (up to 240 contractions per day). In addition, various strategies were used in this study to reveal the correct and real effect of PFMT (verification and teaching of contractions by digital palpation, supervision and follow-up, and adding static and dynamic endurance to traditional strength training alone).

Miller et al. [40] tested the effect of the Knack maneuver before and during coughing in women with stress UI and reported a significant reduction in UI severity after just 1 week. This maneuver, which is also defined as functional training of the pelvic floor muscles, is also recommended to be used not only during coughing or sneezing but also during all daily activities that cause an increase in intra-abdominal pressure [41]. In three studies, within the scope of PFMT, individuals with post-prostatectomy UI were asked to consciously contract their pelvic floor muscles during daily living activities, but no specific results were stated [8, 16, 42]. Therefore, the present study is important as it shows that adding the Knack maneuver to traditional PFMT can further reduce the severity of male incontinence.

Although objective incontinence measures correlate with the individual’s perception of incontinence, they cannot fully explain this perception. In addition, the personal and social perception related to UI is more negative and higher in men compared to women. Therefore, it is recommended to include subjective self-reported outcome measurements in addition to objective outcome measurements in the evaluation of UI. Measurement of the quality of life level is one of the primary outcome measurements [43]. In our study, the score of subjective UI severity and impact of UI on daily life was accepted as the primary outcome measure and assessed with the Turkish-ICIQ-UI SF, which is a widely used and practical scale. At the end of the 8-week intervention period, a greater improvement in quality of life was detected in the groups receiving additional lifestyle recommendations and Knack maneuver training, compared to the group receiving traditional PFMT alone.

In the present study, the standardized 1-h pad test was used for the assessment of objective UI severity. At the end of the 8th week, it was determined that the objective severity of incontinence decreased more in the group that received lifestyle recommendations training compared to the other groups. It is known that PFMT reduces the severity of objective incontinence and improves quality of life [44–46]. However, our results suggest that incorporating the Knack maneuver and lifestyle recommendations into traditional PFMT may lead to greater improvements compared to PFMT alone.

Beyond overall quality of life, UI can affect various sub-domains of quality of life at different levels and PFMT also improve these domains to varying degrees [47]. Therefore, in this study, the effects of interventions on the sub-dimensions of quality of life were also investigated. As a result, compared to other study groups, more improvements were detected in various sub-domains of quality of life in the group that received additional lifestyle recommendations. This finding suggests that adding comprehensive lifestyle recommendations into PFMT may be beneficial in the management of UI following prostatectomy.

The perception of symptom severity is related to individuals’ perception of the importance of their symptoms. Litwin [48] reported that the severity of UI perceived by the patients differs from the severity of UI reported by health professionals. Jonler [49] reported that 71% of men with a median UI value of 46 ml based on a 24-h pad test ignored this level of UI and described their incontinence as “not a problem” or a “very minor problem.” The degree of UI, which is defined as a “mild or non-problematic” condition by one patient, may be defined as “intolerable” by another patient. Perception of improvement is another parameter that may be influenced by personal attitudes, the attitudes of friends, spouses, relatives and clinicians, and expectations from the intervention. Therefore, patient-reported satisfaction/success with intervention is as important as other objective and subjective measures [50]. Accordingly, our study evaluated changes in global impression of UI severity and improvement, along with other outcome measures. By the 8th week, the group receiving lifestyle recommendations reported greater impression of improvements in severity and overall outcomes compared to the other groups.

Compliance with treatment is the key to achieving the desired treatment result [51]. In our study, it is noticeable that while compliance with recommendations regarding addictive substances (tobacco, alcohol, and carbonated drinks) was relatively low in the group receiving lifestyle recommendations, compliance with other recommendations (tea, coffee, water consumption) looks better. In addition, the physical activity level of individuals in this group increased. In our study, the greater improvement in outcome measures in the group receiving lifestyle recommendations compared to other groups may be attributed to a higher rate of compliance with the recommendations. The factors that provide this high compliance with the lifestyle intervention can be listed as the wide scope of the recommendations, the recommendations being given individually in the form of a training session, providing individuals with a written document containing the recommendations, and reminding the individuals of the recommendations during 2-week clinical controls.

### Strengths of the study

UI is a common problem following prostatectomy and, significantly affects quality of life. A key strength of our study is its randomized controlled design, offering new insights into the conservative management of post-prostatectomy UI. PFMT was delivered in accordance with recommended practices, including contraction verification, emphasis on both endurance and strength, and supervised sessions to ensure motivation and high compliance. The study also evaluated the additional effects of the Knack maneuver, a simple and practical approach, when incorporated into PFMT. Furthermore, comprehensive lifestyle recommendations, based on a thorough literature review, were provided and their impact was assessed. Finally, outcome measurements were obtained using widely used instruments with good reliability and validity.

## Study limitations

All interventions were delivered by the same clinicians across each group, and it is important to consider the potential for bias towards one of the approaches. This could be seen as a limitation in our study. To ensure treatment fidelity and mitigate the potential for bias, we implemented a range of strategies, including providing all clinicians with standardized training and protocols to ensure consistent delivery of the interventions. These measures helped reduce bias and maintain consistency in the treatment approach across all participants. Another limitation of our study is that the long-term effects of the study interventions were not demonstrated. In future studies, compliance and response to long-term interventions should be evaluated.

## Conclusion and recommendations

The study results suggest that combining PFMT with Knack maneuver and comprehensive lifestyle recommendations can lead to greater improvements in both objective and subjective severity of UI, overall quality of life, and sub-dimensions of quality of life compared to PFMT alone. In parallel with these results, better results emerge in the combined intervention groups in terms of perceptions of severity and improvement of UI. Long-term results should be investigated in future studies.

In the future, the effects of whole body-oriented exercises (e.g., aerobic exercises) alone or in combination with PFMT on urinary, sexual, and general health can also be investigated in the same population from a more holistic perspective.

## Supplementary Information

Below is the link to the electronic supplementary material.

Supplementary file1 (DOC 221 KB)

## Data Availability

No datasets were generated or analysed during the current study.
